# Bioactive Polymer Composites for 3D-Printed Bone Implants: A Systematic Review

**DOI:** 10.3390/polym18030397

**Published:** 2026-02-03

**Authors:** Anastassiya Khrustaleva, Dmitriy Khrustalev, Azamat Yedrissov, Polina Rusyaeva, Artyom Savelyev, Marlen Kiikbayev, Kristina Perepelitsyna, Vladimir Kazantsev

**Affiliations:** School of Pharmacy, Karaganda Medical University, Gogol Street 40, Karaganda 100008, Kazakhstan; anasteishin_2009@mail.ru (A.K.);

**Keywords:** bioactive polymers, bioactive composites, 3D printing, additive manufacturing, bone tissue engineering

## Abstract

Polymer-based bioactive composites are one of the most rapidly advancing areas in contemporary regenerative medicine. This review aims to identify major trends and knowledge gaps in the development of bioactive polymer composites and examine their translational relevance from a materials design perspective, with a specific focus on synthetic thermoplastic polymer matrices suitable for load-bearing bone scaffold applications and filament-based additive manufacturing. A total of 546 publications spanning 2016–2025 were screened, with 106 selected according to predefined relevance criteria. Bibliometric and content analyses were performed to delineate the primary research trajectories of bioactive composite materials. The results revealed that the majority of studies focused on composites comprising synthetic aliphatic polyesters, primarily polylactic acid (PLA) or polycaprolactone (PCL), reinforced with hydroxyapatite (HA) or bioactive glass (BG), which confer osteoconductivity but rarely achieve multifunctionality. Antimicrobial agents, ion-releasing components, and naturally derived bioactive molecules—associated with biointeractive functionalities and reported effects related to osteogenesis, angiogenesis, and immune modulation—are significantly underrepresented. Fewer than 20% of the investigated studies include in vivo validation, underscoring considerable scope for further preclinical and translational research. This work consolidates current trends in synthetic bioactive polymer composite design and identifies critical directions for future research. The findings of this review provide a structured framework to support the selection of composite fabrication and modification strategies, functional additives, and targeted biological functionalities for next-generation, load-bearing bone tissue engineering materials.

## 1. Introduction

Over the past two decades, additive manufacturing technologies have fundamentally transformed the fabrication of medical devices by enabling the production of highly customized constructs with complex geometries. Among these technologies, three-dimensional (3D) printing has emerged as a cornerstone of personalized medicine, particularly through its capacity to generate anatomically accurate structures tailored to individual patient anatomy [1,2,3]. In the context of polymer-based medical devices, 3D-printing approaches compatible with the processing of synthetic thermoplastics have become especially relevant, as they allow the fabrication of patient-specific implants with controlled architecture and reproducible mechanical performance.

This capability is especially critical in dentistry, craniomaxillofacial surgery, and orthopedics, where defect restoration demands not only mechanical stability but also support for osteogenesis and tissue remodeling [4]. Advances in materials science have shifted the paradigm from inert structural scaffolds to functional composites, in which the polymer matrix serves as a versatile platform for incorporating bioactive agents that synergistically combine mechanical integrity with biological functionality [5]. Biodegradable polymers such as PLA and PCL provide temporary structural support followed by gradual replacement with newly formed tissue [6], whereas non-degradable polymers, including polyetheretherketone (PEEK), are preferred when long-term stability and high mechanical strength are required [7]. Nevertheless, neat polymer matrices rarely achieve an optimal balance of biological and mechanical performance, which has driven intensive research into composite systems.

Bioactive fillers such as hydroxyapatite, β-tricalcium phosphate, and bioactive glass have long been established as osteoconductive components that enhance mineralization and apatite formation [8,9,10]. Concurrently, increasing attention has been directed toward antibacterial agents, including silver, zinc, and copper ions, which are incorporated to mitigate the risk of postoperative infections [11], as well as anti-inflammatory and antioxidant modifiers capable of regulating the cellular microenvironment [12]. Collectively, these developments are converging toward multifunctional materials designed to address structural, osteoinductive, and anti-infective requirements within a single composite system.

Despite substantial progress, existing reviews in this domain remain largely fragmented and narrowly focused. Some publications address only specific polymer matrices or their variants [6,13,14], whereas others concentrate on individual bioactive agents or their mechanisms of action [15]. Comprehensive works that systematically compare integration strategies and biological outcomes across different material–agent combinations are scarce. Moreover, a holistic classification correlating polymer matrix type with bioactive agent characteristics remains lacking, as does quantitative insight into their relative prevalence and reported functional characteristics, which hinders informed material selection in application-specific contexts.

The present systematic review addresses this gap by consolidating contemporary research on bioactive polymer composites intended for bone implants fabricated using 3D-printing technologies applicable to synthetic polymers. In the analyzed literature, studies employing fused deposition modelling (FDM) predominate, while other extrusion-based or specialized additive manufacturing approaches are encountered less frequently and are considered in a comparative context. For clarity and consistency, the terms “3D printing” and “3D-printed” are used throughout this manuscript to refer to FDM-based fabrication, unless explicitly stated otherwise. Materials are categorized according to polymer matrix type, bioactive agent class, and integration methodology. In addition, a descriptive quantitative analysis of the literature dataset is performed to quantify the frequency of different material–agent combinations and to outline prevailing research trajectories. This combined systematic and quantitative approach aims to organize the existing knowledge base and to identify key trends and priorities for future investigations.

## 2. Materials and Methods

This systematic review was conducted in accordance with the PRISMA 2020 guidelines (Preferred Reporting Items for Systematic Reviews and Meta-Analyses) to ensure transparency and reproducibility of the search, screening, and analysis procedures. The PRISMA 2020 checklist is provided as Supplementary Material.

### 2.1. Search Strategy

Literature searches were conducted in the Scopus and Web of Science databases to identify publications published from January 2016 to September 2025. The searches were performed on 10 September 2025. The initial search yielded a total of 546 records, including 250 from Scopus and 296 from Web of Science.

The search strategy combined relevant keywords and Boolean operators as follows:

(TITLE-ABS-KEY (“3D printing” OR “additive manufacturing”) AND TITLE-ABS-KEY (bioactive OR osteoinductive OR antimicrobial) AND TITLE-ABS-KEY (bone OR “bone tissue engineering” OR “bone regeneration”) AND TITLE-ABS-KEY (filament OR filaments OR composite OR “composite material” OR polymer)).

The complete search strategy, including all keywords, Boolean operators, and database-specific syntax, is provided in the Supplementary Material to ensure full reproducibility of the search process.

All retrieved records were exported to the Rayyan software platform (Rayyan Systems Inc., Cambridge, MA, USA; web-based version) for duplicate removal and subsequent screening. After deduplication, 338 unique records remained for further evaluation.

### 2.2. Inclusion and Exclusion Criteria

Studies were included if they met the following criteria: (i) original experimental investigations; (ii) a focus on the development or characterization of bioactive polymer composites intended for the 3D printing of bone implants; (iii) published in the English language.

Studies were excluded if they were review articles or conference abstracts, investigated exclusively metallic or ceramic materials without a polymer matrix, lacked information on bioactive agents, or did not involve 3D printing-based fabrication.

### 2.3. Study Selection

The initial screening of titles and abstracts resulted in the exclusion of 203 records that did not meet the predefined inclusion criteria. The remaining 135 articles underwent full-text assessment, after which 29 studies were excluded due to failure to satisfy the eligibility criteria. Ultimately, 106 original research articles were included in the systematic review. Study screening and selection were performed independently by two reviewers. Any disagreements were resolved through discussion and consensus. No automation tools were used in the screening process.

### 2.4. Data Extraction and Analysis

From each included study, the following data were extracted: polymer matrix type, bioactive agent composition, mechanical properties of the resulting materials, and reported outcomes of in vitro and in vivo biological evaluations.

To facilitate a more accurate and transparent assessment of the strength of evidence across the included literature, the studies were additionally categorized according to the type of experimental model used for biological evaluation. Specifically, studies were classified as based exclusively on in vitro experiments, including in vivo validation, or not reporting explicitly defined in vitro or in vivo biological testing.

For studies incorporating in vivo experiments, a further stratification was applied according to the type of model, including small mammalian models (rats or mice), larger mammalian models (rabbits or sheep), and non-mammalian embryonic models, such as the zebrafish embryo and the chorioallantoic membrane (CAM) assay. This stratification was applied for descriptive and interpretative purposes only and did not influence study eligibility or inclusion decisions.

The extracted data were organized in tabular form and subjected to descriptive quantitative analysis to identify prevalent trends and structural patterns within the literature. No formal risk-of-bias or study quality assessment was performed, as the present work was designed as a descriptive systematic mapping review rather than a clinical effectiveness review or meta-analysis.

### 2.5. PRISMA Flow Diagram

**Figure 1:**
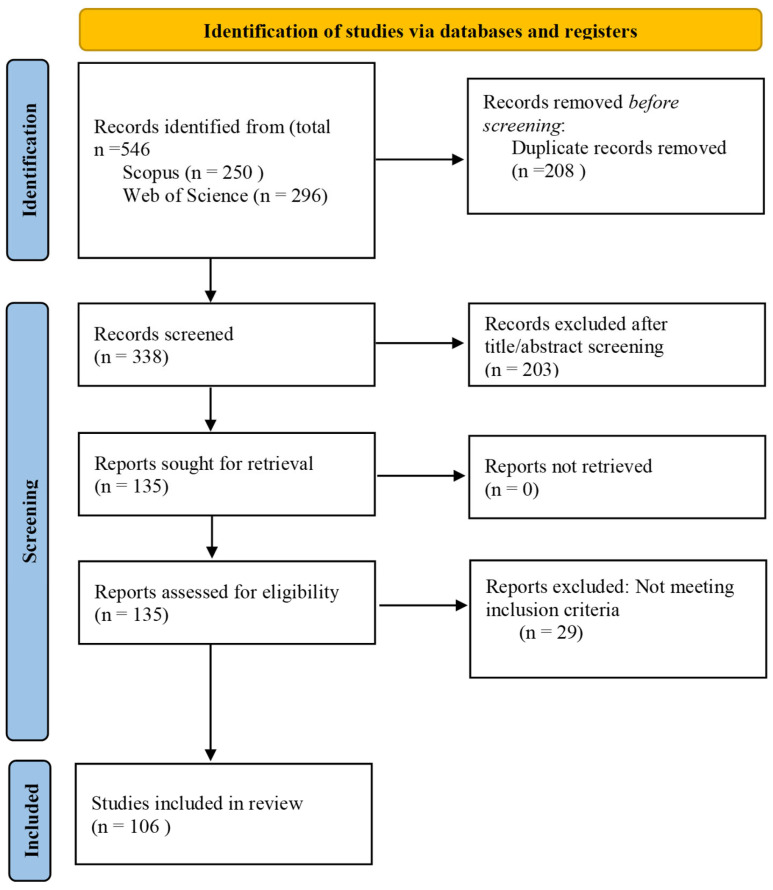
PRISMA flow diagram summarizing the identification, screening, eligibility assessment, and final inclusion of publications in accordance with PRISMA guidelines.

The identification, screening, eligibility assessment, and inclusion process is illustrated in the PRISMA flow diagram (Figure 1).

### 2.6. Descriptive Quantitative Analysis

To enable quantitative comparison and reveal structural patterns in the distribution of investigated materials, descriptive quantitative analysis was performed on data extracted from the 106 selected publications. Parameters were analyzed separately for polymer matrices, bioactive agents, and their combinations.

Polymer matrices were quantified based on the number of publications in which a given polymer served as the primary structural component of the composite, reflecting the distribution of research focus across different matrix types. In cases where copolymers were investigated, the predominant polymer was assigned.

Bioactive agents and their combinations with polymer matrices were tallied by frequency, as several studies employed multicomponent systems or compared multiple additives. Each occurrence of a specific agent (e.g., hydroxyapatite, bioactive glass, ion-releasing additives, or peptides) was recorded independently, even when multiple agents were reported within a single publication. This approach enabled an accurate assessment of the prevalence of individual additive types.

Relative frequencies (%) of each matrix and agent category were calculated with respect to the total number of observations. Distribution charts and a heatmap depicting polymer matrix–bioactive agent combinations were constructed to visualize patterns and intensity of research activity across categories.

All data processing and frequency calculations were conducted using Microsoft Excel, while data visualization was performed using the Datawrapper platform (Datawrapper GmbH, Berlin, Germany; web-based version).

### 2.7. Methodological Scope and Limitations of the Search Strategy

The literature search was restricted to the Scopus and Web of Science databases, which provide extensive coverage of materials science, engineering, and additive manufacturing research. Biomedical databases such as PubMed/MEDLINE and Embase were not included, and therefore some clinically oriented or medically relevant studies may not have been captured.

In addition, no clinical trial registry search was performed, as the scope of this review was focused on preclinical material development and experimental investigations rather than on clinical interventions or trials. These methodological choices reflect the primary aim of the review to analyze material composition, processing strategies, and biofunctional performance of polymer-based composites for 3D-printed bone implants.

## 3. Classification of Bioactive Polymer Composites by Polymer Matrix Type

The selection of the polymer matrix represents a critical determinant of success for bioactive filaments in tissue engineering and regenerative medicine. The matrix governs the mechanical performance, degradation behavior, compatibility with bioactive agents, and suitability for additive manufacturing processes. It influences not only the printability of the filament (e.g., processing temperature, extrusion rate, and layer adhesion) but also key biological responses, including cell adhesion, proliferation, and the osteoinductive or osteoconductive potential of the resulting composite.

A systematic evaluation of the literature reveals two primary classes of polymer matrices: biodegradable and nonbiodegradable. Biodegradable polymers (e.g., PLA, PCL, PGA, and PLGA) predominate in bone tissue engineering owing to their controlled degradation profiles and excellent biocompatibility, positioning them as preferred candidates for resorbable implants and scaffolds. In contrast, non-biodegradable polymers (e.g., PEEK and PET) offer superior mechanical strength, chemical resistance, and dimensional stability—attributes that are particularly advantageous for reconstructing large or load-bearing defects—yet their clinical translation is constrained by the need for surface modification and the incorporation of bioactive additives to enhance osseointegration.

Consequently, the analysis of contemporary bioactive filament research necessitates consideration of the polymer matrix as both the structural backbone and the functional platform onto which bioactive agents are integrated. This section presents a classification of polymer matrices currently employed in filament development for 3D printing, followed by a discussion of their respective advantages, limitations, and prospects for bone regeneration applications.

A comparative overview of the principal polymer matrix classes, including their benefits and constraints, is provided in Table 1.

### 3.1. Polylactic Acid

PLA is the most widely used biodegradable polymer in additive manufacturing for bone regeneration. This thermoplastic polyester, derived from renewable sources such as corn starch or sugarcane, offers excellent biocompatibility, controlled degradation, and favorable processing characteristics for 3D printing [29].

At the molecular level, PLA exists in several stereochemical forms, including poly(L-lactic acid) (PLLA), poly(D-lactic acid) (PDLA), and racemic poly(D,L-lactic acid) (PDLLA). These stereochemical variants differ substantially in crystallinity, thermal behavior, mechanical performance, and degradation kinetics—parameters that are critical for material processability and performance in bone implant applications. Semi-crystalline PLLA generally exhibits higher stiffness and slower degradation due to its higher crystalline fraction, whereas PDLLA is predominantly amorphous and is characterized by lower thermal transition temperatures, improved melt processability, and more homogeneous and accelerated degradation [117].

From the standpoint of intrinsic material properties, PLA provides sufficient initial mechanical support for bone regeneration applications. Reported values of the elastic modulus typically fall within the range of 1.5–2.7 GPa, while the tensile strength is approximately 50–70 MPa, which meets the requirements for temporary load-bearing in resorbable bone scaffolds [27]. Hydrolytic degradation of PLA proceeds via a bulk erosion mechanism involving cleavage of ester bonds, resulting in the formation of lactic acid and low-molecular-weight oligomers that are subsequently metabolized through physiological pathways.

The degradation rate of PLA varies over a wide range depending on stereochemistry, molecular weight, degree of crystallinity, scaffold architecture, and environmental conditions. Under physiological in vitro conditions, an initial decrease in molecular weight of approximately 30–60% is typically observed within the first 3–6 months, while the corresponding mass loss during this period generally remains below 5–10%, reflecting bulk hydrolytic degradation rather than surface erosion. In vivo, complete resorption of PLA-based implants commonly occurs over a period of approximately 6 months to 2 years, whereas more prolonged material persistence, extending up to 3–5 years, has been reported for highly crystalline or high-molecular-weight formulations. In this context, amorphous PDLLA typically loses molecular weight and mechanical integrity more rapidly than semi-crystalline PLLA, which may be advantageous in applications requiring accelerated scaffold resorption synchronized with the rate of new bone formation [117,118].

Despite these advantages, neat PLA exhibits several inherent limitations, including brittleness, low surface hydrophilicity, and biological inertness, which restrict cell adhesion and proliferation. In addition, the accumulation of acidic degradation products during hydrolysis may lead to local pH reduction and inflammatory responses, potentially compromising long-term clinical outcomes.

These limitations are commonly addressed through physicochemical modification, copolymerization, or the development of PLA-based composite systems. One approach involves tuning rheological and biological properties through the incorporation of low-molecular-weight additives and copolymers. For example, the addition of cholecalciferol (vitamin D_3_) reduces the melt viscosity of high-molecular-weight PDLLA, facilitates filament extrusion, and simultaneously enhances osteogenic differentiation [27]. Triblock PLA–PEG–PLA copolymers, characterized by a glass transition temperature of approximately 21 °C and increased hydrophilicity, improve fiber formation via melt electrowriting and enhance cell–material interactions [28].

To enhance biological activity, PLA is frequently formulated into composite systems incorporating bioactive fillers such as HA, bioactive glass, or calcium carbonate [16,17,18,19,42,43]. These components compensate for the intrinsic hydrophobicity and biological inertness of PLA, impart osteoconductive properties, promote the formation of an apatite-like surface layer, and accelerate mineralization. It has been shown that the incorporation of 5–15 wt% HA improves osteoconductivity, supports apatite formation, and increases surface hydrophilicity, whereas higher loadings (>15 wt%) result in particle agglomeration and deterioration of mechanical properties. In particular, Rodovalho et al. [38] demonstrated that PLA/nHA composites containing 10 wt% nanohydroxyapatite achieve an optimal balance between mechanical strength and cellular activity. The addition of 5–20 wt% S53P4 bioactive glass further enhances biomineralization and provides a moderately pronounced antibacterial effect through the release of calcium, sodium, and phosphate ions [17].

Recent strategies extend beyond the incorporation of inorganic components alone and include biomolecules and growth factors. For instance, the use of recombinant BMP-2 in PLA-based scaffolds has been shown to induce a pronounced osteoinductive effect in vivo following implantation [34].

To stabilize local pH during degradation, buffering additives such as low concentrations (≈1 wt%) of bioactive glass or biocompatible carbonates are employed. These additives neutralize acidic degradation products while simultaneously enhancing cell adhesion [25].

In applications requiring prolonged mechanical stability, PLA is often combined with more slowly degrading polymers. In particular, blends of PLA with PCL at weight ratios of 70/30 or 50/50 are regarded as compromise systems balancing stiffness and elasticity while providing controlled degradation behavior [20,23]. The incorporation of up to 15 wt% HA into such blends increases compressive strength to 70–80 MPa, bringing the material closer to the mechanical properties of cortical bone while maintaining high cellular adhesion.

Considerable attention has also been paid to the influence of PLA molecular weight on printability and filament performance. Low-molecular-weight PLA (≈30 kDa) exhibits reduced melt viscosity and improved extrudability but increased brittleness, whereas high-molecular-weight grades require precise temperature control during processing to prevent thermal degradation [27,35].

Organic and carbon-based modifications of PLA are also of interest. Composites incorporating cellulose fibers (UPM Formi-PLA) or nanodiamonds (Udiam-PLA) demonstrate enhanced crystallinity and thermal stability, although their biomedical potential requires further in vitro evaluation [39]. Antibacterial modification of PLA is likewise an active area of research; studies have shown that the incorporation of silver nanoparticles or synthetic antibiotics provides long-term antibacterial activity against *S. aureus* and *E. coli* without significant deterioration of mechanical properties or cytocompatibility [11,36,46,50]. This feature is particularly important for preventing infection-related complications associated with bone implant applications.

In summary, PLA serves as a versatile platform for bioactive filaments and scaffolds. Although inherently limited in osteoinductivity, strategic combination with osteoconductive, antimicrobial, or buffering agents enables precise tuning of mechanical, degradation, and biological performance, making PLA a cornerstone material for multifunctional, resorbable systems in bone tissue engineering.

### 3.2. Polycaprolactone

In addition to PLA, polycaprolactone is one of the most frequently employed biodegradable polymers in tissue engineering and additive manufacturing. Both materials exhibit high biocompatibility and thermoplastic behavior suitable for layer-by-layer fabrication [9]. PCL is distinguished by its low melting temperature (55–60 °C), which minimizes thermal degradation of heat-sensitive bioactive agents and proteins during FDM printing [67,70].

PCL also offers high flexibility, a semicrystalline structure, and slow degradation, with complete loss of mechanical function of PCL-based constructs typically occurring over a period of approximately 2–3 years, making it particularly suitable for implants requiring prolonged structural support [61,69]. More prolonged material persistence has been frequently reported for highly crystalline and high-molecular-weight PCL formulations, in which reduced water uptake and limited chain mobility further slow bulk hydrolytic cleavage, thereby extending functional material integrity beyond the typical 2–3-year resorption window [119,120].

Importantly, PCL undergoes bulk hydrolytic degradation, in which molecular and mechanical degradation precede macroscopic mass loss. Quantitative studies indicate that under physiological conditions the molecular weight of PCL typically decreases by approximately 20–50% over periods ranging from several months to two years, while the corresponding mass loss during the same timeframe generally remains below 1–10%. In vitro investigations have reported mass losses of approximately 4–5% over 6–8 months, accompanied by a deterioration of mechanical properties exceeding 40%, whereas long-term in vivo studies of 3D-printed PCL implants demonstrate minimal mass loss (<1%) after one year followed by gradual resorption of approximately 10% after two years [119,121,122].

However, this inherently slow resorption profile limits the applicability of neat PCL in tissues characterized by rapid regeneration. To address this limitation, researchers commonly blend PCL with faster-degrading polymers such as PLA, PHBV, or gelatin, thereby creating hybrid systems with tailored degradation gradients and improved temporal matching between scaffold resorption and tissue formation [59,71].

Mechanically, PCL strikes a favorable balance between flexibility and strength, which is advantageous for highly porous scaffold architectures. The Young’s modulus of neat PCL typically ranges from 0.35 to 1 GPa [11], while compressive strength values between 1 and 8 MPa have been reported [61], which are comparable to those of trabecular bone.

A major drawback of PCL is its pronounced hydrophobicity (water contact angle ≈ 78°), which can impair cell adhesion and proliferation [65,76]. To overcome this limitation, various surface and bulk modification strategies have been proposed, including the incorporation of hydrophilic copolymers such as poly(phenylene sulfone) (PPSu) [58], surface coatings with proteins or polysaccharides (e.g., gelatin, collagen, alginate, or polyvinyl alcohol) [59,62], and immobilization of natural biopolymers cross-linked with genipin [74]. These approaches improve surface wettability and enhance cell–matrix interactions, thereby promoting extracellular matrix deposition.

The molecular weight significantly influences the mechanical performance and degradation kinetics. Low-molecular-weight PCL (14 kDa) tends to be brittle and mechanically weak, whereas medium (45 kDa) and high (80 kDa) variants provide an optimal combination of strength, flexibility, and resorption rates for bone engineering applications [59,60,68,69].

Given the intrinsic bioinertness of PCL, current efforts focus on imparting osteoconductive and osteoinductive properties without compromising printability or mechanical integrity. The most common strategy involves adding inorganic particles such as HA or β-TCP [75,79,94]. These fillers improve surface wettability, promote apatite layer formation, and accelerate osteoblast differentiation [85,87]. BG incorporation similarly enhances biomineralization while reinforcing porous architectures [88,96,97].

More advanced approaches include piezoelectric phases such as barium titanate (BaTiO_3_), which generate local electric fields under mechanical loading and thereby stimulate osteogenesis [60,76].

PCL composites have also been widely investigated as drug delivery platforms. Silver nanoparticles increase the elastic modulus to ∼1 GPa and provide sustained antimicrobial activity against *Staphylococcus aureus* and *Escherichia coli* [11]. Encapsulation of gentamicin or halloysite nanotubes enables prolonged release and improved infection control [63].

Emerging fillers such as AK/MNT enhance bioactivity, although excessive loading can reduce melt viscosity and impair print quality [82].

Collectively, these developments position PCL as a versatile platform for multifunctional filaments that combine structural support, osteoinduction, and antimicrobial functions. Its biocompatibility, flexibility, and extensive modification potential make it well suited for long-term implants and complex anatomical constructs. Nonetheless, its hydrophobicity, limited stiffness, and slow degradation necessitate careful engineering—through hydrophilic blending and bioactive filler incorporation at the material level, combined with optimization of scaffold design parameters, including pore architecture and molecular characteristics—to achieve the desired balance between mechanical stability and regenerative performance.

### 3.3. Poly(lactic-co-glycolic acid)

PLGA is a biodegradable copolymeric matrix whose physicochemical properties can be broadly tailored by adjusting the ratio of lactide and glycolide units. In contrast to PLA homopolymers, PLGA exhibits increased hydrophilicity and generally accelerated degradation kinetics, which makes it particularly attractive for temporary bone constructs intended for the early stages of regeneration. The ability to fine-tune resorption behavior enables synchronization of mechanical support with the sequential phases of osteogenesis and bone remodeling [98].

With respect to degradation behavior, PLGA undergoes bulk hydrolytic cleavage of ester bonds, typically following a staged process in which molecular weight reduction precedes substantial mass loss [123]. Quantitative studies demonstrate that degradation kinetics are strongly composition-dependent: glycolide-rich formulations (e.g., PLGA 50:50 or 65:35) represent the fastest-degrading systems and may undergo near-complete degradation within approximately 1–2 months under physiological conditions, whereas lactide-rich copolymers (e.g., PLGA 75:25 or 85:15) exhibit slower degradation extending over 4–6 months or longer [123,124]. This pronounced tunability of degradation behavior enables precise temporal control of scaffold resorption and release profiles but also necessitates careful consideration of copolymer composition and local pH effects in biomedical applications.

In the context of additive manufacturing, the principal limitation of PLGA arises from the combination of moderate mechanical strength and the thermal sensitivity of incorporated bioactive components, which significantly restricts the applicability of conventional melt-based FDM printing. Consequently, most 3D-printed PLGA-based bone scaffolds are fabricated using extrusion-based solution or low-temperature techniques, including anti-solvent printing, low-temperature rapid prototyping, and paste extrusion followed by lyophilization [99,100]. These approaches enable the formation of highly porous architectures at near-ambient temperatures while preserving the functionality of thermolabile biomolecules.

As a polymer matrix, PLGA exhibits limited intrinsic osteoactivity and therefore requires targeted functionalization. The most common strategy involves the incorporation of inorganic fillers such as hydroxyapatite or β-tricalcium phosphate, which compensate for the biological inertness of the polymer and provide an osteoconductive surface [98,99]. Further enhancement of the biological response can be achieved through the inclusion of ionic components, particularly magnesium, which has been shown to activate osteogenic and angiogenic signaling pathways and modulate inflammatory responses [102].

Another important direction in the development of PLGA-based matrices is their application as carriers for bioactive molecules and genetic constructs. Gene-activated PLGA scaffolds containing adenoviral vectors encoding BMP-2 have demonstrated pronounced osteoinductive effects, highlighting the potential of PLGA as a platform for localized and controlled delivery of growth factors [100]. In such systems, the polymer matrix plays a predominantly regulatory rather than load-bearing role by defining the spatial and temporal release of biologically active signals.

A promising strategy for improving the processability of PLGA involves its combination with other polyesters, such as poly(3-hydroxybutyrate-co-3-hydroxyvalerate) (PHBV). In these blends, PLGA can act as a modifier of rheological behavior and a regulator of degradation kinetics, while PHBV provides structural stability. When combined with calcium phosphate phases, such hybrid systems exhibit a favorable balance between printability, mechanical performance, and osteogenic potential [101].

Overall, within additively manufactured bone systems, PLGA should be regarded primarily as a functional matrix with controllable and relatively rapid degradation rather than as a load-bearing material. Its composition-dependent resorption profile distinguishes PLGA from more slowly degrading polyesters such as PLA or PCL, positioning it as a material of choice for applications focused on biological regulation, early-stage osteogenesis, and controlled delivery of therapeutic agents rather than long-term mechanical fixation.

### 3.4. Other Biodegradable Polymer Matrices

In addition to aliphatic polyesters and non-degradable high-temperature thermoplastics, a range of alternative polymer matrices has been investigated for the additive manufacturing of bone implants and scaffolds that do not fall directly within the classical PLA or PCL systems. These materials are characterized by distinct degradation mechanisms, mechanical behavior, and modes of biological interaction and are primarily employed to address specialized clinical requirements, including delayed resorption, prolonged retention of mechanical function, enhanced elasticity, or functional adaptation of scaffold architecture to specific clinical scenarios.

This group includes polyester–ether multiblock copolymers, most notably poly(ethylene oxide terephthalate)/poly(butylene terephthalate) (PEOT/PBT). These segmented biodegradable polymers exhibit slower degradation rates and reduced medium acidification compared to aliphatic polyesters and are predominantly used as structurally stable matrices in extrusion-based (FDM-like) bone scaffolds. The principal limitation of PEOT/PBT is its low intrinsic bioactivity, which is typically addressed through composite modification. It has been demonstrated that the incorporation of high loadings of nano-hydroxyapatite yields scaffolds with mechanical properties comparable to cancellous bone; however, the biological response is governed mainly by ionic exchange and surface mineralization rather than direct osteoinduction [105]. In addition, antibacterial PEOT/PBT-based implants have been developed in which bioactive molecules are incorporated via inorganic carriers, ensuring their stability during extrusion-based 3D printing and enabling controlled release independent of polymer matrix degradation [107].

An alternative strategy is represented by PHA-containing systems, particularly blends of poly(3-hydroxybutyrate) (PHB) with PLA. PHB is characterized by high crystallinity and favorable biocompatibility; however, its application in FDM printing is limited by brittleness and a narrow processing window, necessitating blending with PLA and the use of plasticizers [104]. For PHB/PLA matrices modified with β-tricalcium phosphate, it has been shown that the biological response is largely governed by surface free energy and wettability rather than solely by the chemical composition of the material. This finding highlights the importance of simultaneous optimization of material composition and FDM processing parameters in the design of bioactive bone scaffolds [104]. Such systems may be considered as alternatives to PLA in applications requiring a stiffer matrix while retaining biodegradability.

Other polymer matrices include biodegradable elastomeric polyurethanes, particularly segmented poly(ether urethanes) (SPEU). Their primary advantage lies in high deformability and tunable mechanical properties; however, their relatively low elastic modulus limits their use in load-bearing bone defects, directing these materials mainly toward non-load-bearing or partially load-bearing applications. To increase stiffness, composite SPEU-based systems incorporating stiffer biodegradable polymers and bioactive fillers have been proposed. While these approaches improve the mechanical performance of extrusion-printed scaffolds, they also increase printing complexity and necessitate trade-offs between bioactivity and technological reproducibility [106].

A distinct category is represented by polyurethane–polyester matrices with shape-memory behavior, which are applied in bone scaffolds. In systems based on blends of thermoplastic polyurethane and polycaprolactone (TPU/PCL), programmable shape recovery enables minimally invasive implantation and geometric adaptation of the scaffold to the defect site. In such materials, bioactivity is primarily achieved through surface modification strategies, such as polydopamine coating, without compromising shape-memory performance or the mechanical integrity of the scaffold [103].

Overall, alternative polymer matrices extend the capabilities of additive manufacturing for bone implants beyond PLA- and PCL-based systems. However, their practical implementation requires precise tailoring of material composition, structure, and functional properties in accordance with the expected mechanical loading and intended clinical application.

### 3.5. Non-Biodegradable Polymer Matrices

Nondegradable polymer matrices comprise high-performance thermoplastics that maintain long-term stability under physiological conditions. Unlike biodegradable systems, these materials provide permanent mechanical support, making them valuable for load-bearing or permanent implants. However, publications over the past decade have revealed significantly fewer studies on nondegradable polymers for 3D printing and their bioactive modification than their biodegradable counterparts. This disparity arises from both the processing challenges of high-temperature polymers and the need to overcome their inherent bioinertness through functional additives.

Among the reviewed studies, polyaryletherketones (PAEKs), particularly polyetheretherketones (PEEKs) and polyetherketones (PEKs), have received the most attention as promising candidates for additively manufactured load-bearing bone implants [110,112,114]. These polymers exhibit high stiffness (Young’s modulus 3–4 GPa), excellent chemical resistance, and radiolucency, positioning them as viable alternatives to titanium alloys in craniomaxillofacial reconstruction and orthopedic applications [10,113].

Despite these advantages, nondegradable PAEK-based polymers are intrinsically bioinert and hydrophobic, which limits direct bone bonding and necessitates additional strategies to promote osseointegration.

PEEK (e.g., VESTAKEEP^®^ 2000 UFP) remains the most extensively investigated matrix owing to its favorable combination of mechanical strength, chemical inertness, radiolucency, and sterilization stability [109]. To overcome the intrinsic biological inertness of PEEK in bone implant applications, composites incorporating HA, strontium- or zinc-doped HA (SrHA, ZnHA), or carbonate-substituted HA (cHA) have been developed, resulting in markedly improved cell adhesion and surface apatite formation [8,111]. The resulting composites display tensile strengths of 47.9–75 MPa and elastic moduli of 2.8–4.2 GPa—values closely matching those of cortical bone—making them suitable for structural and load-bearing applications [108,112].

Surface treatments such as plasma activation or ion implantation further increase the hydrophilicity and osteoinductive potential without compromising the bulk mechanical properties [113,114].

Other members of the PAEK family, including polyetherketone (PEK) and polyetheretherketoneketone (PEKK), are also under active investigation. These polymers share similar chemical backbones but offer enhanced thermal stability and stiffness. Kruse et al. [113] demonstrated that both FDM-printed PEEK and SLS-processed PEK provide excellent mechanical strength and radiolucency, indicating that PAEK-based systems are compatible with multiple additive manufacturing routes relevant to bone implant fabrication. PEKK, in particular, combines structural robustness with biocompatibility but requires additional physicochemical modifications to elicit strong cellular responses [10]. Its higher glass transition temperature and improved osteoblast adhesion further expand the thermomechanical design space within the PAEK family.

Although nondegradable matrices excel in durability and load-bearing capacity, their permanence necessitates surgical removal in cases of complications. High-density and hydrophobic surfaces also hinder cell and vascular infiltration, requiring optimized porosity and hybrid strategies—such as macroporous architectures that retain sufficient mechanical competence.

Overall, PEEK and related PAEK derivatives remain highly promising for permanent and load-bearing bone reconstruction. Current development trends emphasize the combination of exceptional mechanical stability with tailored bioactivity through the use of osteoinductive fillers, nanostructured surfaces, and multilayer hybrid designs.

## 4. Classification of Bioactive Agents

This section focuses on the classification of bioactive agents incorporated into polymer composites for 3D-printed bone substitutes. These agents are introduced to impart specific biological functions, such as osteoconduction, osteoinduction, antimicrobial activity, or controlled ionic and molecular release. In this review, bioactive agents are systematically classified according to their primary biological function, as summarized in Table 2.

As shown in the data, calcium–phosphate compounds dominate bioactive fillers, with HA and BG being the most prevalent owing to their excellent biocompatibility, compositional similarity to the mineral phase of bone, and pronounced osteoconductivity. β-Tricalcium phosphate is employed considerably less frequently, yet it has comparable osteoinductive potential through the controlled release of calcium and phosphate ions. Antimicrobial agents, peptides, proteins, and naturally derived additives are reported only in isolated studies.

### 4.1. Hydroxyapatite (Ca_10_(PO_4_)_6_(OH)_2_)

Hydroxyapatite constitutes the primary inorganic component of bone and is widely incorporated as an osteoconductive agent in polymer composites for 3D-printed bone implants [125]. Its chemical and structural similarity to native bone apatite confers excellent biocompatibility, promotes osteogenic cell adhesion and proliferation, and facilitates calcium–phosphate layer formation in physiological environments [126].

HA serves as a nucleation template for apatite deposition and subsequent mineralization, establishing a robust implant–bone interface. It also modulates local ion exchange by releasing Ca^2+^ and PO_4_^3−^, thereby stimulating osteogenesis and upregulating markers such as ALP, RUNX2, and OCN [66,69].

Both natural and synthetic HA variants have been reported. Mocanu et al. incorporated bovine-derived HA into PLA at 0–50 wt%, reducing the water contact angle from ∼70° to 21° and enhancing cell adhesion; however, loadings above 30 wt% induced particle agglomeration and compromised composite homogeneity [16].

Synthetic HA, produced via chemical precipitation, hydrothermal synthesis, sol–gel methods, or microwave-assisted methods, allows precise control over morphology, particle size, and crystallinity. Owing to its relatively high solubility, low-crystallinity nano-HA (∼39%) accelerates biomineralization and promotes osteogenic cell adhesion [16]. Conversely, excessive crystallinity limits ion exchange, and apatite layer formation is critical for osseointegration.

Particle size and morphology significantly influence bioactivity and mechanical performance. Both micro-HA (1–40 µm) [37,66,94] and nano-HA (20–346 nm) [9,57,69,71] have been explored, with direct comparisons demonstrating that nano-HA yields more uniform Ca–P layers and superior osteoblast proliferation and differentiation [9,65], whereas micro-HA supports macroporosity and vascularization but is prone to agglomeration at high loadings [16,23,65]. The optimal HA content typically ranges from 10–20 wt% to maintain homogeneity and mechanical integrity; concentrations exceeding 30 wt% often cause clustering and internal stress [16,23,26,37].

At the same time, HA has also been investigated at very low contents (<1 wt%) not as a reinforcing filler but as a bioactive modifier of the polymer–cell interface. Under these conditions, low-dose HA was shown to improve osteoblast adhesion and viability while exerting minimal influence on scaffold architecture and bulk mechanical properties [59].

Chemical substitution further enhances biological performance. Carbonate-substituted HA (CHA) closely mimics biological apatite (carbonate content 4–8 wt%) by partial replacement of PO_4_^3−^ or OH^−^ with CO_3_^2−^, reducing crystallinity, increasing solubility, and accelerating Ca–P layer formation [8,26]. In PLA composites, 5–20 wt% CHA (<90 µm) improves ASC spheroid adhesion and viability [18,26]. In PEEK matrices, CHA supports 500–800 µm porosity and biomineralization, although >30 wt% CHA increases brittleness and impairs cytocompatibility [8].

Ion-doped variants have also been widely investigated. Strontium-substituted HA (SrHA) promotes osteoblast proliferation while inhibiting osteoclast activity [22,111,115], and zinc-substituted HA (ZnHA) combines osteogenic stimulation with mild antibacterial effects through Zn^2+^ release [111,115].

Hybrid systems combining HA with other bioactive phases show synergistic benefits. Coincorporation with borosilicate BG enhances osteogenic differentiation via Ca^2+^ and Na^+^ release, although excessive glass (>20 wt%) reduces cell viability [37].

In summary, HA remains the most prevalent osteoconductive filler in 3D-printed composites owing to its biomimetic composition and ability to upregulate osteogenic markers. Nanosized and ion-substituted forms at 5–20 wt% provide the strongest osteoinductive response while preserving mechanical performance. Key limitations include particle agglomeration at high loadings and inconsistent ion release profiles.

### 4.2. Bioactive Glasses

BGs are silicate-, phosphate-, and borosilicate-based materials capable of triggering biomineralization and forming strong chemical bonds with bone. Their bioactivity arises primarily from the controlled release of Ca^2+^, Si^4+^, and PO_4_^3−^ ions, which drive surface apatite-like layer formation. In 3D-printed polymer composites, BG serves as both an osteoconductive filler and an osteoinductive filler, enhancing osteogenic cell adhesion, proliferation, and differentiation. This dual role positions the BG as a critical component when rapid biomineralization and osseointegration are needed [127].

The most established composition remains 45S5 Bioglass^®^ (46.1% SiO_2_, 24.4% Na_2_O, 26.9% CaO, 2.6% P_2_O_5_), which was originally developed by Hench. Its incorporation into polymer matrices promotes dose-dependent apatite deposition and surface hydrophilicity [49]. Sustained Ca^2+^ release in the nanomolar range over extended immersion correlates with enhanced surface mineralization. At 5 wt% (D50 = 4.3 µm), controlled Ca^2+^ release (0.03–1 nmol/µL over 168 h) confirms mineralization stimulation [28].

The related S53P4 composition (53% SiO_2_, 23% Na_2_O, 20% CaO, and 4% P_2_O_5_) exhibited slower ion exchange kinetics, resulting in gradual Ca^2+^ release (0.35 nmol/µL over 7 days) and robust Ca–P layer formation [17,19]. It supports mesenchymal stromal cell adhesion and metabolic activity but typically requires additional cues (e.g., BMP-2) for pronounced osteoinduction.

More complex formulations, such as 13–93 BG (Na_2_O–K_2_O–MgO–CaO–SiO_2_–P_2_O_5_), incorporate MgO and K_2_O to further stimulate osteogenesis via Mg^2+^ and SiO_4_^4−^ release [36]. High BG loadings (up to 50 wt%), however, can increase the pH to 8.5 and impair cell viability.

To improve osteoinductive potential and biocompatibility, surface and ionic modifications of conventional BG have been widely explored. These strategies modulate ion release kinetics, enhance osteogenic differentiation, and mitigate cytotoxicity at elevated Si and Ca concentrations. Current efforts also confer antibacterial, anti-inflammatory, and angiogenic properties [10,29,64].

Surface coatings provide one effective route. The gold coating of 45S5 particles regulates ion release while reinforcing mechanical stability, although excessive thickness suppresses Ca^2+^ and Si^4+^ release and compromises bioactivity [29]. Polydopamine (PDA) functionalization similarly improves cell adhesion and uniform apatite deposition in PLCL/BG composites through the combination of chemical and topographical cues [49,74].

Ionic doping with Sr^2+^, Zn^2+^, Ce^3+^, or Ag^+^ enables targeted cellular responses. Strontium doping upregulates ALP and RUNX2 expression while inhibiting osteoclast resorption [21,74]. Zinc-doped BG (e.g., A2Zn5 in PCL) combines osteoconduction with antimicrobial activity [88]. Cerium incorporation reduces inflammatory responses and supports regeneration under postimplantation conditions [10].

Hybrid modifications pairing doping with biomolecular functionalization further enhance performance. Zn-doped BG combined with osteogenin, for example, delivers concurrent antimicrobial protection and osteogenic stimulation [64].

Compared with conventional 45S5 or S53P4, advanced mesoporous (MBG) and nanostructured bioactive glasses markedly increase the specific surface area and ion-exchange capacity. This structural hierarchy accelerates apatite formation and activates osteogenic signaling pathways, shifting MBG from purely osteoconductive to genuinely osteoinductive behavior [55,72,94,97].

Dendritic MBG (SiO_2_–CaO–P_2_O_5_) synthesized via sol–gel methods exhibit 2–10 nm pores and high surface reactivity, driving rapid Ca^2+^, Si^4+^, and PO_4_^3−^ release. Loadings of 5–30 wt% enhance osteoblast adhesion, proliferation, and HA crystallization while serving as both a mechanical reinforcement and an ionic donor [72].

Strontium-doped MBG (MBG_SG_10%Sr) achieves near-complete Sr^2+^ release within 7 days, significantly increasing osteogenic activity [21]. Simvastatin-loaded MBG simultaneously exploits glass osteoconductivity and drug-mediated activation of the BMP2 and Wnt/β-catenin pathways, resulting in accelerated mineralization and osteoblast differentiation [33].

In summary, bioactive glasses—from classic 45S5 to mesoporous, doped, and hybrid variants—represent a versatile group of inorganic agents that deliver combined osteoconductive, osteoinductive, and antimicrobial effects. Advances in doping, porosity control, and release kinetics now enable precise tuning of bioreactivity in 3D-printed filaments, establishing the BG as a cornerstone component in bone regenerative engineering.

### 4.3. β-Tricalcium Phosphate

Compared with HA and BG, β-tricalcium phosphate appears less frequently in the reviewed studies but remains an important resorbable calcium–phosphate ceramic with high biocompatibility and controlled biodegradability. Its defining feature is gradual dissolution in physiological fluids, releasing Ca^2+^ and PO_4_^3−^ ions that support apatite-like structure formation. This property makes β-TCP particularly suitable for applications requiring synchronization between material degradation and new bone formation. Compared with HA, β-TCP results in faster resorption; compared with BG, β-TCP results in weaker osteoinductivity, necessitating careful evaluation of its role within polymer composites [31].

The influence of the β-TCP content and particle size on bioactivity and mechanical performance has received considerable attention. The incorporation of 5–20 wt% β-TCP into PLA filaments for FDM printing enhanced biocompatibility and osteoconductivity by promoting apatite formation in simulated body fluid (SBF). The 10–15 wt% range yielded the optimal balance between strength and bioactivity, whereas higher loadings increased brittleness [45]. Similar findings were reported with 0–15 wt% β-TCP, which drove active biomineralization and uniform Ca/P distribution (by energy-dispersive X-ray spectroscopy (EDS)) but did not significantly enhance the osteogenic differentiation of human mesenchymal stem cells (hMSCs), confirming its primarily osteoconductive action [101].

β-TCP also modifies surface physicochemical properties. The addition of 13 wt% β-TCP (3–15 µm) to PHB/PLA composites reduced the water contact angle from 74° to 61° and increased the surface energy, which was correlated with improved Saos-2 osteoblast-like cell adhesion and proliferation [104]. Comparable effects were observed at lower loadings (5 mg/g, <50 µm), although precise dosing optimization was emphasized [75]. Thus, β-TCP functions not only as an ionic source but also as a topographical modifier that regulates cell adhesion through microroughness and surface polarity.

A key advantage of β-TCP over other inorganic fillers is its ability to buffer acidic degradation products from polyesters (PLA, PLGA), thereby maintaining physiological pH and microenvironment stability. Zhang et al. [98] demonstrated that a 1:4 β-TCP:PLGA ratio reduced medium acidity while enhancing osteoconductivity. The resulting scaffolds featured macropores (∼490 µm) and micropores (2–15 µm) that supported efficient cell and vascular infiltration. Even low loadings (2.5 wt%) of β-TCP and CaCO_3_ stabilized the pH and improved PLA bioactivity, although mechanical reinforcement remained limited [32].

Recent work has focused on nanoscale β-TCP, which offers a greater specific surface area and reactivity. Nanoβ-TCP has been shown to strengthen 3D-printed scaffolds and improve osseointegration through enhanced bone contact, albeit with potential risks of accelerated degradation and local pH shifts [40].

Direct comparisons with HA and BG highlight distinct profiles. Unlike HA, β-TCP undergoes rapid bioresorption, making it preferable when swift replacement by the host bone is desired. Relative to BG, it exhibits superior compatibility with polyester matrices and greater chemical stability but releases fewer biologically active ions (e.g., Si^4+^, Sr^2+^), resulting in lower osteoinductive capacity. Its primary role is therefore to provide an osteoconductive framework while buffering degradation byproducts.

Overall, β-TCP serves as a versatile yet supplementary bioactive agent that enhances biomimetic performance and creates a favorable osteogenic microenvironment. Its effectiveness is maximized when it is combined with other active components (HA, ionic dopants, or growth factors) or when it is employed in nano- or mesoporous forms.

### 4.4. Mineral and Ion-Releasing Agents

Mineral- and ion-releasing agents represent a broad and functionally diverse class of bioactive components that extend beyond conventional calcium phosphate ceramics and bioactive glasses. The incorporation of ions such as Ca^2+^, Mg^2+^, Si^4+^, and Zr^4+^ provides an effective strategy to stimulate osteogenesis by participating in bone mineral formation and modulating osteoblast signaling pathways.

Among these systems, magnesium-containing polymer composites have attracted particular attention due to their combined mechanical, biological, and immunomodulatory effects. For example, the addition of magnesium sulfate to PLGA (PLGA-2Mg) increased mechanical stiffness while promoting macrophage polarization toward an anti-inflammatory M2 phenotype, indicating that Mg^2+^-containing systems can indirectly enhance osteogenesis through immune regulation beyond conventional osteoconduction [102].

Importantly, recent studies have demonstrated that 3D-printed Mg-containing polymer composite scaffolds can progress beyond preclinical validation. Advanced in vivo animal studies have been reported for biodegradable Mg–PLGA–TCP porous scaffolds [128], while more recent human clinical evaluation has confirmed their translational potential. In a multi-center randomized controlled trial, Mg–PLGA–TCP composite scaffolds significantly accelerated early bone fusion compared to a commercial β-TCP control, confirming their clinical relevance in human patients [129].

Recent efforts have also focused on calcium–magnesium silicates, including monticellite (CaMgSiO_4_), akermanite (Ca_2_MgSi_2_O_7_), baghdadite (Ca_3_ZrSi_2_O_9_), and apatite–wollastonite (AW). Controlled release of Ca^2+^, Mg^2+^, Si^4+^, and Zr^4+^ from these minerals activates osteoblastic differentiation and accelerates calcium–phosphate phase deposition. Even low loadings (5–15 wt%) in PLA or PCL matrices significantly upregulate osteogenic markers such as ALP, RUNX2, and COL1 [61,68,82].

Piezoelectric osteoactive fillers, particularly barium titanate (BaTiO_3_), have also attracted considerable interest. Under mechanical loading, BaTiO_3_-containing composites generate local electric microfields that enhance osteoblast differentiation [60,76]. Despite this potential, its high dielectric constant and possible Ba^2+^ release raise long-term biocompatibility concerns, necessitating surface modification for safe integration with biodegradable polymers.

Calcium- and phosphate-based glasses further serve as tunable sources of Ca^2+^ and Si^4+^. The incorporation of calcium–phosphate glass (G5) into PLA yielded sustained Ca^2+^ release (up to 12.5 mmol over 21 days), stimulating osteogenesis while upregulating the angiogenic factor VEGF [43]. This dual action highlights the capacity of ionic stimulation to concurrently support osteogenesis and vascularization—critical processes in bone defect healing.

### 4.5. Antimicrobial Agents

Only a limited subset of the reviewed studies incorporated antibacterial agents into bioactive polymer composites for 3D printing, indicating that antimicrobial functionalization remains underexplored. Most publications prioritize osteoconductive and osteoinductive performance, whereas infection prevention—a leading cause of implant failure—receives attention only sporadically.

Infection prophylaxis is a critical development axis for 3D-printed bone substitutes, as bacterial biofilm formation on implant surfaces triggers chronic inflammation and osteolysis and often requires revision surgery [130,131]. Current strategies embed antimicrobial agents directly within filament formulations to achieve sustained, localized release while preserving mechanical integrity and biocompatibility.

Among inorganic agents, silver-based compounds dominate [132]. Ag^+^ ions exert bactericidal effects through multiple mechanisms, including membrane disruption, inhibition of electron transport, DNA damage, and reactive oxygen species generation. Contemporary research has focused less on proving efficacy and more on optimizing the concentration, morphology, and incorporation method to balance antimicrobial potency with cytotoxicity.

Afghah et al. reported that 1–2.5 wt% silver nitrate (AgNO_3_) in polymer matrices dose-dependently inhibited *E. coli*, *S. aureus*, *P. aeruginosa*, and *C. albicans*, with zones of inhibition reaching 15.3 ± 1.4 mm at 2.5 wt% silver nitrate. The released Ag^+^ levels (4.5–7.5 mg/kg) remained below the cytotoxic thresholds, demonstrating clinical feasibility without compromising cell viability [22]. Notably, this work established that conventional silver salts—rather than nanoparticles—can be safely encapsulated in thermoplastic filaments while retaining activity.

In contrast, silver nanoparticles (AgNPs) provide prolonged ion release and enhanced contact killing. Radhakrishnan et al. [11] reported that 20–150 nm AgNPs in PCL yielded inhibition zones of up to 20.4 mm against *E. coli* and a 2.2-log reduction within 24 h. Porous scaffolds (>300 µm) retained their osteoblast infiltration capacity, with no cytotoxicity observed up to 15 mg Ag/g composite.

Certain bioactive glasses, particularly S53P4, exhibit intrinsic antimicrobial activity without additional drugs [133,134]. The incorporation of 5–20 wt% S53P4 suppresses both anaerobic and aerobic bacterial growth while maintaining mechanical performance [19]. This activity stems from ion exchange (Na^+^, Ca^2+^, P^5+^, Si^4+^) and localized pH elevation, effectively inhibiting the growth of multidrug-resistant strains such as MRSA and MRSE [19]. Strontium doping of mesoporous glasses further enhances antibacterial efficacy alongside osteogenesis [74].

Organic and hybrid systems are also under active investigation [36]. Broad-spectrum antibiotics—including gentamicin, tobramycin, ciprofloxacin, and vancomycin—retain activity after extrusion and printing temperatures up to 180 °C, delivering bactericidal doses over two weeks [12,50,63,89]. Gentamicin-loaded PLA filaments, for example, release 52–104 µg on day 1 and 2.7–28 µg through day 14, exceeding minimum bactericidal concentrations [63]. Combining gentamicin with Zn^2+^ ions extends duration and potency [36].

The use of natural and synthetic biocidal molecules is gaining traction. Carvacrol immobilized on PLA substrates forms controlled-release biocidal coatings with broad-spectrum activity [41]. ε-Polylysine (EPL) achieves up to 92% inactivation of *E. coli* and *S. aureus*, although rapid burst release (∼80% within 24 h) limits long-term protection [66].

Emerging alternatives include photosensitive black phosphorus nanosheets (BP-NSs), which generate reactive oxygen species under near-infrared irradiation, resulting in ∼90% bacterial inactivation [113]. Antimicrobial peptides (e.g., Tet213, Y-Tet213, and Mel4) offer broad activity against *S. aureus*, *P. aeruginosa*, and fungi [83,114]. DOPA conjugation improves surface anchoring and in vivo stability, although peptide retention during polymer degradation requires further study.

In summary, antimicrobial functionalization of bioactive filaments remains limited in scope but highly promising. Silver-, zinc-, and strontium-containing glasses, along with antibiotics such as gentamicin and vancomycin, have synergistic antimicrobial and osteoinductive effects, laying the foundation for the development of next-generation multifunctional 3D-printed composites for bone regeneration.

### 4.6. Other Osteoactive Agents and Combined Approaches

In addition to conventional inorganic fillers such as HA, β-tricalcium phosphate, and bioactive glass, emerging osteoactive agents are being explored for their ability to modulate cellular, biochemical, and immune pathways in bone regeneration. These strategies involve the incorporation of biomolecular cues, natural organomineral particles, functional coatings, pharmacological agents, and electroactive components into 3D-printed composites. The resulting multifunctional materials have synergistic effects, including concurrent stimulation of osteogenesis and angiogenesis, regulation of inflammation and infection, and optimization of scaffold biomechanics.

#### 4.6.1. Proteins, Peptides, and Signaling Molecules

Proteins, peptides, and low-molecular-weight bioregulators constitute an emerging class of bioactive agents for 3D-printed bone implants. Their appeal lies in direct modulation of osteogenesis, angiogenesis, and antimicrobial defense, enabling precise tuning of the regenerative response.

Recent review literature highlights a growing research interest in low-molecular-weight bioactive small molecules incorporated into polymer-based matrices for bone regeneration, particularly within PLA- and PCL-based composite systems [135,136]. Plant-derived compounds, including flavonoids and phenolic acids, are discussed as biochemical modulators of osteogenic and angiogenic pathways [135]. In this context, Zhang et al. reported a PLGA/β-tricalcium phosphate scaffold functionalized with the plant-derived small molecule icaritin and mesenchymal stem cell-derived secretome, demonstrating enhanced osteogenic differentiation and bone regeneration in vivo. This example illustrates how molecular and biological cues can be integrated into polymer–ceramic scaffolds to complement the osteoconductive role of the mineral phase [98]. In parallel, representative experimental studies have reported the integration of molecules such as icariin, salvianolic acid, and puerarin into polymer composite scaffolds to achieve localized biochemical stimulation [137,138,139].

Cholecalciferol (VD_3_) exemplifies small-molecule regulators with dual functional roles [27]. Its incorporation into PDLLA reduces melt viscosity, facilitates filament extrusion, and upregulates osteogenic markers such as ALP and OCN. However, due to its thermal sensitivity, the applicability of VD_3_ in conventional FDM processing remains limited, and the lack of in vivo validation currently constrains its translational relevance.

Fusion and adhesion peptides represent a more advanced category. The immobilization of osteo- and angiogenic peptides on PLGA/HA scaffolds enhanced MC3T3-E1 osteogenic differentiation and supported in vivo defect regeneration [99]. Challenges include strong dose dependence, susceptibility to proteolysis, and the need for precise surface loading. Cassari et al. [112] partially addressed these limitations via the use of a retro-inverso peptide (D2HVP), which exhibited greater proteolytic stability and improved osteoblast adhesion, underscoring the importance of chemical modification for peptide performance.

Hybrid approaches employing extracellular matrix components (e.g., collagen–fibrin hydrogels or decellularized MSC-derived matrix) provide excellent cell adhesion and robust angiogenic responses but suffer from inferior mechanical properties compared with structural polymers, limiting their utility in critical-size defects [67].

Growth factors, particularly BMP-2, remain the most potent osteoinductive cues. Low-dose, controlled-release BMP-2 markedly accelerates bone formation [34,73], a critical advantage given known complications at high doses. However, achieving stable, prolonged, and noninflammatory delivery remains unresolved. Gene-activated constructs generated via adenoviral BMP-2 vectors sustain long-term expression but raise immunogenicity and safety concerns [100]. In vivo findings further underscore the necessity of controlled growth factor delivery. In the study [140], the application of rhBMP-2 in combination with an allogeneic bone graft accelerated early osteoregeneration, but at later stages resulted in heterogeneous outcomes and episodes of excessive bone resorption due to disrupted osteoblast–osteoclast balance. These observations highlight the critical importance of achieving a dose-regulated and sustained release profile of BMP-2 in 3D-printed implants.

Antimicrobial peptides (AMPs), such as Mel4, effectively prevent bacterial colonization, which is a vital feature of implants [114]. However, reduced osteoblast mineralization in their presence highlights an inherent trade-off between bactericidal activity and cytocompatibility.

#### 4.6.2. Organomineral and Natural-Derived Agents

Organominerals and naturally derived agents form a distinct class of osteoactive fillers that follow a biomimetic strategy by replicating the structure and composition of native bone.

Prominent examples include calcium-rich natural structures such as marine mollusk shells and coral skeletons. Abalone shell particles (ASPs), containing Ca, Mg, collagen, and chitin, were incorporated into PCL scaffolds and created a bioactive microenvironment that promoted osteoblast proliferation and enhanced extracellular matrix mineralization [78]. A similar bioinspired approach employs oyster shell powder (OSP); its addition to biopolymer matrices markedly increased alkaline phosphatase activity and accelerated bone matrix mineralization [91].

Decellularized bone extracellular matrix represents another avenue [84]. When integrated into polymer constructs, it preserves the native bone architecture and provides optimal cues for cell infiltration. These systems bridge natural and synthetic materials by combining inherent biological activity with the mechanical robustness of polymeric scaffolds.

#### 4.6.3. Functional Coatings and Carbon Nanostructures

Surface functionalization provides an effective route to enhance the biocompatibility and osteoactivity of 3D-printed polymer constructs without compromising their bulk mechanical properties. These coatings enable targeted modulation of cell behavior and mineralization.

Polydopamine (PDA) is a widely studied biomimetic coating that improves surface hydrophilicity and roughness, thereby promoting cell adhesion and proliferation [49,74,92,103]. PDA has been shown to upregulate osteogenic markers (BMP-2, collagen I) and facilitate calcium–phosphate layer formation, establishing a favorable microenvironment for osseointegration [92].

Carbon nanotubes (CNTs) are incorporated primarily for mechanical reinforcement and to support cell proliferation [110]. One example combined BG with CNTs as a coating on polylactic acid [42]. However, excessive CNT concentrations can induce cytotoxicity and oxidative stress, necessitating careful dose optimization and thorough biocompatibility assessment.

## 5. Quantitative Trend Analysis of Published Literature

To identify dominant trends in the development of bioactive polymer composites for 3D-printed bone implants, a statistical analysis was performed on 106 publications selected according to systematic review criteria. The analysis examined the distribution of studies by polymer matrix type, bioactive agent category, and their combinations. These findings provide insight into the current landscape and emerging directions in additive manufacturing for bone tissue engineering.

### 5.1. Distribution of Publications by Polymer Matrix Type

Statistical evaluation revealed a clear predominance of biodegradable thermoplastics in the literature on bioactive composites for 3D printing, with PCL and PLA together accounting for over 80% of the reported systems (Table 3).

**Figure 2:**
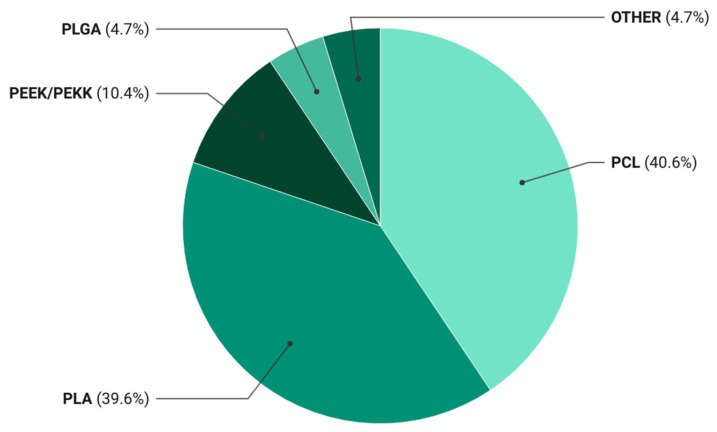
Distribution of polymer matrices used in 3D-printed bioactive composites. Pie chart showing the relative frequency (%) of polymer matrix types reported in the 106 publications included in the systematic review.

For a visual representation of the matrix usage frequency, the data are presented in Figure 2.

The data revealed that PCL- and PLA-based materials accounted for 40.6% and 39.6% of the publications, respectively. This distribution reflects a persistent preference for these matrices as standardized research platforms that ensure result comparability and reproducible processing parameters in bioactive filament development. The dominance of PCL and PLA has led to the establishment of a de facto model system in the field, driven not only by their biocompatibility and tunable degradation but also by well-established extrusion and printing protocols.

This concentration accelerates the accumulation of robust datasets yet simultaneously constrains innovation by limiting the exploration of alternative matrices with distinct mechano-biological profiles. The performance of PCL- and PLA-based composites relies heavily on bioactive and reinforcing fillers, as neat polymers provide only baseline strength and functionality. Their prevalence thus underscores both their technological maturity and their dependence on secondary phases to confer osteoconductive, osteoinductive, antimicrobial, or ion-regulatory properties.

PEEK/PEKK systems appear far less frequently, representing approximately 10% of studies. Despite their superior mechanical and chemical stability, their adoption is restricted by high melting temperatures, lack of biodegradability, and the need for specialized equipment. These polymers have been investigated primarily for permanent, load-bearing applications—such as spinal or craniomaxillofacial implants—whereas current research emphasis has shifted toward resorbable, tissue-integrating systems designed for gradual replacement by host bone.

Overall, the statistical analysis indicates that research on bioactive composites operates within two principal paradigms:

- A model paradigm centered on biodegradable polyesters (PLA, PCL) that serve as versatile platforms for evaluating bioactive agents and modifications.
- An engineering paradigm focused on non-resorbable polyaromatic thermoplastics (PEEK, PEKK) intended for durable, mechanically demanding constructs.

The marked shift toward the former reflects a broader trend in biomaterials science: the transition from inert structural substitutes to actively interacting systems capable of both mechanical support and biological regulation through controlled degradation and functional component release. Thus, the current literature confirms a sustained trajectory toward next-generation biodegradable matrices characterized by adaptability, functional versatility, and seamless integration into complex tissue environments.

### 5.2. Distribution of Publications by Bioactive Agent Type

Statistical analysis of the 106 reviewed publications enabled quantitative assessment of the frequency of different bioactive agents in polymer composites for 3D-printed bone substitutes. Because several studies incorporated multiple agents or combinations, counts were based on mentions, yielding a total of 127 instances distributed across nine functional categories (Table 4).

**Figure 3:**
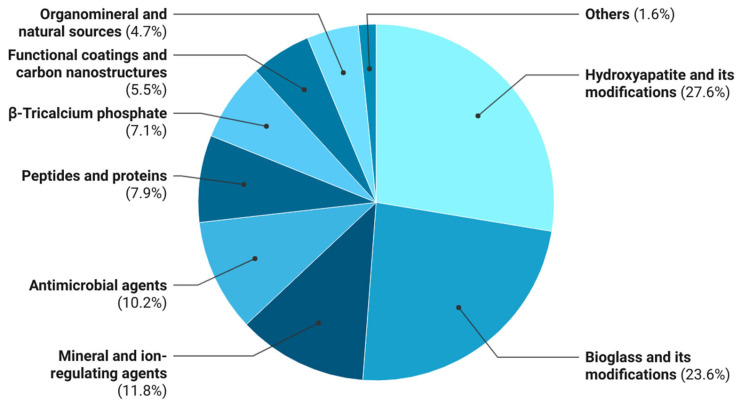
Distribution of bioactive agents used in 3D-printed polymer composites. Relative frequency (%) of bioactive agent classes reported in the analyzed literature.

For a visual representation of the distribution across bioactive agent categories, the data are presented in Figure 3.

As shown in Table 4, inorganic osteogenic fillers dominate the literature, with hydroxyapatite and its derivatives (27.6%), bioactive glass and its variants (23.6%), β-tricalcium phosphate (7.1%), and ion-releasing agents (11.8%) collectively accounting for 70.1% of mentions. These materials remain the primary osteoconductive and, in some cases, osteoinductive components, mimicking the inorganic phase of bone. In most studies, however, they are incorporated into monofunctional systems primarily focused on promoting osteogenesis, without providing additional biological functionalities.

Antimicrobial agents, including silver compounds, antibiotics (gentamicin, vancomycin, ciprofloxacin), and functional nanoparticles, account for 10.2% of bioactive-agent mentions. Their incorporation aims to mitigate postimplantation infection risk; however, the majority of such work is confined to in vitro models, and combined osteoinductive–antimicrobial systems remain underexplored.

Multicomponent composites (e.g., PLA/HA/vancomycin or PCL/bioactive glass/Zn) offer integrated action—simultaneously promoting osteogenesis, reducing the bacterial load, and modulating inflammation. Their low representation highlights the need to shift design paradigms from monofunctional toward hierarchically organized systems in which each component contributes synergistically.

Peptides and proteins (7.9%), including growth factors (BMP-2, VEGF) and antimicrobial peptides (Mel4), represent an emerging class of signaling agents capable of directing cell differentiation and vasculogenesis. However, their integration into thermoplastic matrices is constrained by thermal processing challenges in FDM, which compromise the native biomacromolecule structure. Progress in this area will require low-temperature incorporation strategies and hybrid inorganic carriers.

Natural organomineral sources (4.7%), such as corals, eggshell, bone ash, and marine exoskeletons, also remain underexplored. These materials exhibit biomimetic compositions and contain diverse ions (Ca^2+^, Mg^2+^, Sr^2+^, PO_4_^3−^) that participate in osteogenic regulation. The limited publications suggest that the potential of sustainable, naturally derived fillers has not yet been fully realized. Further development could yield environmentally compatible, biomimetic, and cost-effective next-generation composites.

Key Findings

- Current research on bioactive composites for 3D printing focuses on monofunctional osteogenic systems based on hydroxyapatite, bioactive glass, and β-tricalcium phosphate.
- Multicomponent composites that integrate osteogenic, antimicrobial, and anti-inflammatory functions have been investigated sparingly but represent the most promising trajectory.
- Natural calcium- and phosphorus-containing materials possess considerable potential as sustainable biomimetic agents, but their application demands standardization and detailed evaluation of polymer–matrix interactions.

### 5.3. Combinations of Polymer Matrices and Bioactive Agents

Analysis of polymer matrix–bioactive agent pairings revealed patterns that reflect current material design strategies for additive manufacturing. Whereas the previous sections addressed the individual frequencies of matrices and agents, the focus here is on their co-occurrence, which defines the functional profile of the composite.

**Figure 4:**
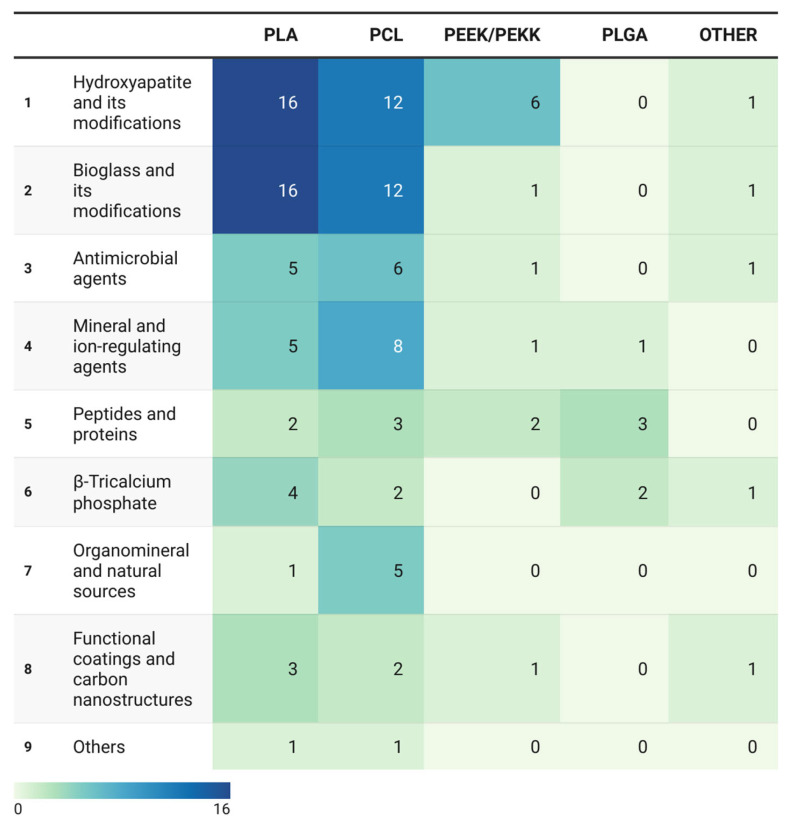
Heatmap of co-occurrence frequencies between polymer matrices and bioactive agent categories. Heatmap illustrating the frequency of matrix–agent combinations across 106 included studies (*n* = 127 mentions).

The distribution is visualized as a heatmap (Figure 4), where color intensity corresponds to the frequency of each matrix–agent combination.

The data demonstrate pronounced clustering around technologically mature systems—predominantly PLA and PCL paired with osteoinductive fillers—while multifunctional combinations and naturally derived agents remain at the periphery of research interest.

The heatmap color scale, ranging from light green to dark blue, indicates the research activity intensity for each matrix–agent pair, revealing marked asymmetry in the distribution of efforts across polymer types.

The most intense regions (dark blue) cluster at intersections of PLA and PCL with conventional ceramic agents—HA and BG. These pairings constitute the core of current research on the use of bioactive composites for additive manufacturing.

The high frequency of these combinations stems from the pronounced osteoconductivity and compositional similarity of HA and BG to bone minerals. However, their dominance also reflects a constrained research paradigm. The peak values occur for PLA/HA (16 cases), PLA/BG (16 cases), PCL/HA (12 cases), and PCL/BG (12 cases). These systems reliably promote apatite formation and osteoblast activity in vitro; however, in vivo validation remains limited. Moreover, they predominantly represent monofunctional composites focused solely on osteogenesis, without addressing additional biological challenges such as antimicrobial or anti-inflammatory protection.

This pattern is particularly evident compared with antimicrobial agent pairings (5 for PLA, 6 for PCL, 1 for PEEK, and 1 for other matrices), which account for only 10% of the total mentions. The disparity underscores a research emphasis on osteoconductive strategies at the expense of therapeutically relevant functions, despite their clinical importance.

Thus, future advancements in PLA- and PCL-based composites should shift from increasing HA/BG variations toward multifunctional systems integrating osteoconductive, antimicrobial, and bioregulatory components tailored for clinical translation.

Notably, PLA combined with natural or organomineral agents appears extremely rarely (≤1 mention), indicating an under-exploration of this topic. These findings position PLA as a chemically and functionally conservative platform requiring the strategic expansion of biomimetic filler options.

In contrast, the PCL heat profile exhibited a broader and more balanced distribution. In addition to ceramics, PCL shows greater incorporation of ion-releasing agents (8 mentions) and natural/organomineral sources (5 mentions).

The PEEK/PEKK and PLGA matrices display lighter to moderate cyan–green shades, reflecting specialized applications. PEEK/PEKK pairings form moderate-intensity zones restricted primarily to HA and peptide/protein additives, which is consistent with their use as mechanically robust platforms necessitating surface functionalization for bioactivity.

Conversely, PLGA exhibits low-intensity but localized clusters with peptides, proteins, and β-TCP, which aligns with its established role in controlled biomolecule delivery rather than mechanical reinforcement.

Key Findings

- Current research concentrates on a narrow set of combinations (PLA/PCL with HA and BG), providing a stable foundation but restricting functional diversity.
- Pairings involving antimicrobial, ion-releasing, and protein/peptide agents remain sporadic and demand improved methods for controlled integration and printing.
- Although natural calcium- and phosphorus-containing sources offer substantial biomimetic potential, they are virtually absent from systematic additive manufacturing studies.
- The most promising trajectory lies in designing multicomponent systems that integrate multiple biofunctions—osteoinductive, antimicrobial, and anti-inflammatory—within a single polymer matrix.

## 6. Stratification of Included Studies by Level of Biological Validation

**Figure 5:**
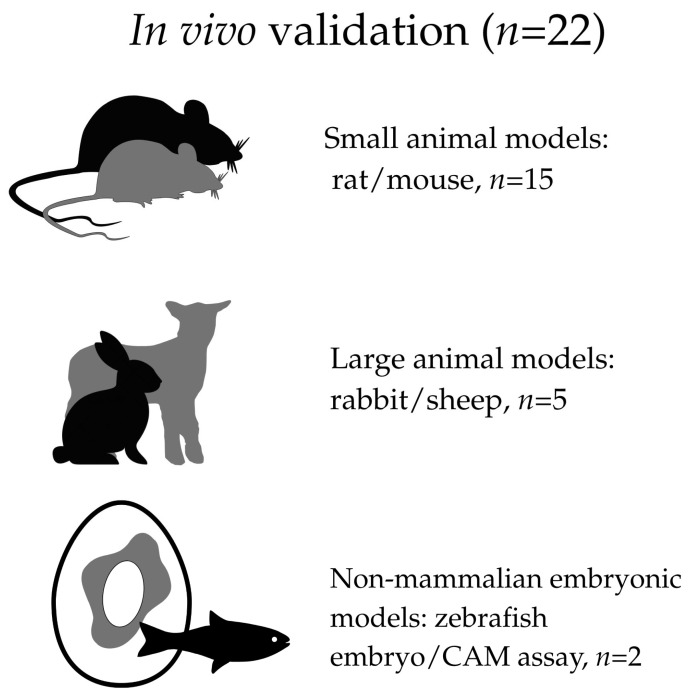
Schematic distribution of in vivo models.

The assessment of the reported biological validation revealed substantial heterogeneity in the levels of experimental evidence supporting the investigated material systems. The distribution of biological validation levels in the analyzed literature is summarized in Table 5, while the stratification of in vivo studies is presented in Figure 5.

Of the 106 studies included in the present systematic review, the majority were based exclusively on in vitro evaluation (*n* = 67), predominantly involving short-term assays of cell viability and osteogenic differentiation. In vivo validation was performed in 22 studies, whereas 17 publications did not report explicitly defined in vitro or in vivo biological testing.

Additional stratification of the in vivo studies (*n* = 22), as presented in Figure 5, demonstrated that small mammalian models, primarily rats or mice, were most frequently employed (*n* = 15). Large mammalian models, such as rabbits or sheep, were used in a limited number of studies (*n* = 5). In addition, two studies employed non-mammalian embryonic models, including zebrafish embryos and the chorioallantoic membrane (CAM) assay (*n* = 2).

This quantitative distribution indicates that, despite the active expansion of research activity in the field of bioactive polymer composites for 3D-printed bone implants, the majority of material systems remain at an early stage of biological and translational validation. This aspect should be taken into consideration when interpreting their potential for clinical translation.

It is evident that results obtained from short-term in vitro tests are not equivalent in translational relevance to data derived from in vivo models; however, this difference reflects not the intrinsic quality of the materials themselves but rather the current stage of their experimental validation. In this context, the predominance of in vitro studies in the literature should be viewed as indicative of early stages of composite development and optimization, whereas the more limited number of studies incorporating in vivo validation points to a gradual transition of certain developments toward more advanced stages of preclinical evaluation.

## 7. Translational and Regulatory Considerations

Despite the rapid expansion of research in the field of bioactive polymer composites for 3D printing of bone implants, their translational readiness is determined not only by the magnitude of osteogenic or antimicrobial effects but also by a set of practical and regulatory factors. These include the safety of the applied concentrations, formulation reproducibility, compatibility with clinically relevant sterilization methods, and the feasibility of scalable manufacturing in compliance with Good Manufacturing Practice (GMP) requirements. Such a gap between experimental efficacy and clinical applicability has been repeatedly emphasized in studies devoted to bioactive polymer systems for bone regeneration [26,84].

Analysis of the included studies demonstrates that the composite systems reported in the literature differ substantially in terms of practical and regulatory maturity. Translationally closer systems are those characterized by simple and predictable formulations, based on clinically well-studied polymer matrices and osteoconductive fillers. This category includes classical polymer–ceramic composites based on PLA/HA and PCL/HA, for which apatite formation in simulated body fluid (SBF), improved cell adhesion and viability, and, in some cases, in vivo validation have been repeatedly demonstrated. Their translational proximity is associated with the use of hydroxyapatite as a regulatorily well-understood bioactivation pathway, a minimal number of components, and the absence of pharmacologically or genetically active payloads [20,26,45,48,65,86].

A similar rationale applies to PLA–BG and PCL–BG systems, including those based on bioactive glasses such as 45S5 and 52S at moderate concentrations (typically on the order of 10–20 wt%). These materials rely on the clinically accepted concept of ionic release and apatite-like layer formation and are also capable of partially neutralizing acidic degradation products of PLA without the incorporation of pharmaceutical agents [17,19,29,70]. Likewise, high-temperature clinical polymers, most notably PEEK, modified with hydroxyapatite or related calcium phosphate phases, demonstrate enhanced bioactivity while maintaining mechanical and thermal stability, rendering PEEK/HA and PEEK/CHA systems conceptually close to clinical implants [8,103,109].

At the same time, a substantial proportion of contemporary studies focus on multifunctional composites incorporating antibacterial ions (e.g., Ag^+^), osteogenic dopants (Sr^2+^, Zn^2+^), and low-molecular-weight bioactive compounds. Although such systems often exhibit pronounced antibacterial, osteogenic, or immunomodulatory effects in vitro, their translational readiness is largely constrained by narrow safe concentration windows and a strong dependence of the biological response on composition and dosage. For silver-containing composites, a clear dose-dependent effect has been demonstrated: at concentrations of approximately 1–2.5 wt%, acceptable bone cell viability is maintained, whereas higher loadings result in pronounced cytotoxicity [11].

A similar pattern is observed for Zn- and Sr-doped hydroxyapatites and bioactive glasses, where positive effects on osteogenesis are achieved only under strictly controlled ion release, while excessive concentrations may retard apatite formation or exert cytostatic effects [64,109,115]. For bioactive glasses, an additional risk factor is local pH alteration resulting from intensive ionic exchange. In several composite formulations, high glass-phase loadings were associated with a shift in pH toward alkaline values (in some cases up to ~8.5), which is potentially unfavorable for bone tissue cells and highlights the necessity of optimizing both the concentration and composition of the glass phase.

The incorporation of low-molecular-weight bioactive compounds, such as vitamin D_3_, statins, antimicrobial peptides, or plant-derived osteoactive molecules, further complicates the assessment of translational potential due to initial burst-release effects, environment-dependent release kinetics, and well-documented antiproliferative effects at elevated local concentrations [27,98,99].

Nanostructured additives, including carbon nanotubes and nanoscale hydroxyapatite, are also characterized by concentration-dependent trade-offs. Particle agglomeration at increased loadings leads to reduced printability, diminished cell adhesion, and, in some cases, cytotoxic effects [69,110]. Additional risks are associated with accelerated degradation of low-molecular-weight polymer matrices in the presence of high mineral filler contents, which may be accompanied by local acidification and decreased cell viability [69].

Antimicrobial polymer composites incorporating synthetic antibiotics warrant separate consideration. For vancomycin-containing systems, the absence of acute cytotoxicity based on metabolic and membrane integrity assays has been demonstrated within an approximate range of 1–20 wt% antibiotic, with values around 10 wt% considered a compromise between antimicrobial efficacy and maintenance of cellular activity [50]. Similarly, gentamicin-loaded composites (2–5 wt%) did not exhibit signs of direct cytotoxicity in vitro [63,89]. However, these systems are characterized by pronounced initial burst release during the first 24 h, potentially resulting in high local drug concentrations. While such release profiles may be clinically justified in the treatment of infection-complicated defects, they indicate a limited therapeutic window and underscore the importance of controlling release kinetics. Accordingly, antibiotic-loaded systems should be regarded as promising provided that further optimization is undertaken.

An additional, yet rarely discussed, aspect of the translational readiness of bioactive polymer composites is their compatibility with clinically relevant sterilization methods. Despite the critical role of sterilization in the translational pathway of 3D-printed bone implants, this parameter is mentioned in only a limited number of studies within the analyzed body of literature (approximately 14 out of 106). The most frequently reported approach is ethanol treatment at various concentrations [11,41,91,100,103], sometimes combined with ultraviolet irradiation [72,92,102]. These methods should be regarded primarily as laboratory disinfection rather than true sterilization, as they do not ensure compliance with regulatory requirements for implantable medical devices.

A smaller number of studies report the use of ethylene oxide sterilization, one of the few methods potentially compatible with clinical practice for thermosensitive polymer systems [47,50,61]. Notably, only one of these studies evaluated residual sterilant levels in accordance with ISO 10993 requirements, with no exceedance of permissible limits detected [50]. Isolated reports also describe the application of alternative low-temperature methods, such as oxygen plasma sterilization [27] and treatment with a 2% peracetic acid solution [31].

Finally, scalability and compatibility with GMP-compliant manufacturing represent another major and largely underestimated limitation of the clinical translation of bioactive polymer composites. The most widely applied technology is FDM, which has been used to fabricate scaffolds based on PLA, PCL, PEEK, PLLA, and PLGA, including systems containing ceramic fillers (hydroxyapatite, β-tricalcium phosphate, bioactive glasses) and functional additives. The widespread adoption of FDM reflects its technological simplicity, geometric reproducibility, and compatibility with clinically relevant thermoplastics.

From a translational perspective, filament-based FDM printing demonstrates the highest degree of compatibility with GMP-compliant manufacturing. This process can be implemented in closed production lines with controlled extrusion and printing parameters, thereby facilitating process validation, quality control, and scalability. An additional argument supporting the translational relevance of FDM is its existing use in the manufacturing of medical devices.

In contrast, techniques such as screw-assisted extrusion, robocasting, and indirect or multistep hybrid technologies involving post-print functionalization offer greater compositional flexibility but are generally implemented in open systems and are characterized by increased variability in material properties and final product performance. The presence of numerous manual operations and the dependence of final characteristics on post-processing conditions significantly complicate standardization, batch-to-batch reproducibility, and implementation within GMP-compliant production.

Taken together, the presented data demonstrate that the translational relevance of bioactive polymer composites is determined not by the maximization of functional effects but by a balance between biological activity, safety, and technological feasibility. This consideration is critical when interpreting their realistic potential for clinical translation.

## 8. Conclusions

This systematic review provides a comprehensive assessment of the current landscape of bioactive polymer composites for 3D-printed bone implants. An analysis of 106 studies revealed that, despite substantial advances in additive manufacturing and established polymer–mineral strategies, research remains heavily concentrated on conventional combinations of biodegradable matrices—primarily PLA and PCL—with HA or BG. These systems offer reliable processability, predictable degradation, and robust in vitro biocompatibility but predominantly function as osteoconductive scaffolds, with limited capacity to address the broader spectrum of biological processes involved in bone regeneration.

A clear shift in research focus is observed from monofunctional composites toward multifunctional, biointeractive systems aimed at addressing multiple biological pathways, including osteogenesis, angiogenesis, immune modulation, and infection control. Emerging directions include multi-ion release profiles (Mg^2+^, Sr^2+^, Si^4+^), peptide and protein functionalization, polydopamine-based coatings, carbon nanostructures, natural organomineral additives, and stimuli-responsive (photo- or piezoactive) materials. These approaches have been increasingly reported to influence cellular behavior, signaling pathways, and coordinated tissue responses within experimental settings.

Nevertheless, a marked imbalance persists in the literature. Mineral fillers dominate current composite designs, whereas antimicrobial agents, immunomodulatory components, and biomolecular cues remain significantly underrepresented despite their potential relevance for clinical applications. In addition, many emerging strategies demonstrate encouraging in vitro outcomes but lack sufficient in vivo validation, limiting accurate assessment of their translational relevance. This gap highlights the need for more integrated design strategies that simultaneously consider osteogenesis, vascularization, infection prevention, and immune regulation.

Despite ongoing progress, standardized material design frameworks, harmonized biological evaluation protocols, and expanded preclinical investigations remain limited. Key unresolved challenges include insufficient emphasis on immunomodulation, angiogenic stimulation, antimicrobial performance, and long-term remodeling dynamics. Strategic optimization of polymer matrices combined with mineral, ion-modulating, and biomolecular additives, together with multimodal surface functionalization, represents a key direction for advancing bioactive composite design.

Overall, this review indicates that the field is progressively moving beyond conventional composite formulations toward next-generation smart biomaterials designed to interact with the complex microenvironment of bone repair. Achieving this transition will require close interdisciplinary collaboration among materials scientists, biologists, pharmaceutical engineers, and clinicians to support the development of 3D-printed implants with improved predictability and functional integration in bone regeneration.

## Figures and Tables

**Table 1:** Primary classes of polymer matrices used in bioactive composites for 3D printing.

Polymer Matrix	Key Advantages	Limitations	References
***Biodegradable polymers***			
**Polylactic acid** and its copolymers	High stiffness and elastic modulus; excellent biocompatibility and predictable degradation. Good printability with dimensional accuracy; strong compatibility with mineral and antibacterial fillers; extensive regulatory and clinical data.	Brittleness and low impact strength; local pH drop during degradation; limited ductility; requires functionalization for enhanced bioactivity.	[16,17,18,19,20,21,22,23,24,25,26,27,28,29,30,31,32,33,34,35,36,37,38,39,40,41,42,43,44,45,46,47,48,49,50,51,52,53,54,55,56,57]
**Polycaprolactone**	High ductility, flexibility, and impact resistance; low melting temperature. Slow, uniform degradation; excellent compatibility with mineral and antimicrobial additives; consistent porosity and morphology in FDM printing.	Low stiffness; prolonged resorption time; less established regulatory pathway; mechanical properties sensitive to crystallinity.	[9,11,12,58,59,60,61,62,63,64,65,66,67,68,69,70,71,72,73,74,75,76,77,78,79,80,81,82,83,84,85,86,87,88,89,90,91,92,93,94,95,96,97]
**Poly(lactic-co-glycolic acid)**	Tunable degradation rate via copolymer ratio; high biocompatibility. Effective platform for drug encapsulation and controlled release; well-suited for multifunctional bioactive systems.	pH decrease during degradation; reduced mechanical strength; shrinkage and warping during printing; unsuitable for load-bearing applications.	[98,99,100,101,102]
**Other polymer** matrices (TPU/PCL, PEOT/PBT, PHB/PLA, SPEU/PHBV)	Broad mechanical spectrum from highly elastic (TPU) to rigid engineering copolymers (PEOT/PBT). Property tuning by blending with PLA or PCL; support complex architectures and functional gradients.	High variability in properties; lack of universal printing parameters. Low strength in soft systems; slow degradation of certain compositions; limited clinical validation.	[103,104,105,106,107]
***Non-biodegradable polymers***			
**Polyetheretherketone**	Mechanical properties comparable to cortical bone; chemical and sterilization resistance. Radiolucency with no MRI/CT artifacts; established clinical safety record; amenable to surface functionalization.	Nondegradable; high processing temperature; challenging filler incorporation; inherently low osteoinductivity without modification.	[8,10,108,109,110,111,112,113,114,115,116]

**Table 2:** Principal groups of bioactive agents used in polymer composites for 3D printing.

Bioactive Agent Type	Primary Functions	Effects on 3D-Printed Constructs/Bone Regeneration	Representative References
**Hydroxyapatite** (HA), SrHA, ZnHA, CHA	Osteoconductivity; Ca–P layer formation; enhanced biocompatibility	Improved mechanical strength; increased cell adhesion and mineralization; accelerated early osteogenesis	[8,9,16,18,20,21,22,23,26,31,35,37,38,45,48,51,54,57,59,65,66,69,71,79,81,85,86,87,94,105,108,109,111,115,116]
**Bioactive glass** (BG), MBG	Osteoinduction; apatite formation; ionic remodeling	Apatite layer formation; enhanced implant integration; stimulation of angiogenesis	[10,17,19,21,25,28,29,30,33,37,42,44,49,53,55,56,64,70,73,74,76,80,88,90,93,95,96,97,106]
**β-Tricalcium phosphate** (β-TCP), Ca_3_(PO_4_)_2_	Resorbability; Ca^2+^/PO_4_^3−^ source; bone remodeling	Osteoblast stimulation; increased mineral density; synchronization of degradation and regeneration	[9,31,32,40,45,89,98,101,104]
**Mineral and ion-releasing additives** (Mg^2+^, Sr^2+^, Mn^2+^, Zn^2+^, BaTiO_3_, etc.)	Modulation of cellular signaling; enhanced osteogenesis; ion exchange	Upregulation of osteogenic markers; accelerated mineralization; improved integration	[24,32,43,47,52,60,61,68,76,80,82,83,87,102,113]
**Antimicrobial agents** (AgNPs, AgNO_3_, Zn^2+^, antibiotics, natural phenols)	Antibacterial activity; biofilm suppression	Reduced infection risk; prevention of inflammation; maintained implant sterility	[11,12,36,41,46,50,51,58,63,66,89,107,113]
**Organomineral and natural materials** (collagen, chitosan, abalone shell, etc.)	Biomimicry of extracellular matrix; improved adhesion; support of proliferation	Enhanced biocompatibility; organized matrix formation; increased hydrophilicity	[48,59,77,78,84,91]
**Proteins and peptides** (BMP-2, RGD, osteoinductive fragments)	Activation of osteogenic differentiation; signaling regulation	Intensified osteogenesis; accelerated defect healing; guided regeneration	[27,34,67,73,83,98,99,100,112,114]
**Functional coatings and carbon nanostructures** (CNT, PDA)	Enhanced adhesion; mechanical reinforcement; surface roughness; conductivity	Modulation of cellular response; increased osteoinduction; improved strength and functionalization area	[20,49,74,92,103,110]

**Table 3:** Distribution of publications across polymer matrix categories.

Polymer Matrix	Number of Publications	Percentage of Total
PCL	43	40.6%
PLA	42	39.6%
PEEK/PEKK	11	10.4%
PLGA	5	4.7%
Other	5	4.7%

**Table 4:** Frequency distribution of bioactive agent mentions.

Bioactive Agent Category	Number of Mentions	Percentage of Total (*n* = 127)
Hydroxyapatite and its modifications	35	27.6%
Bioactive glass and its modifications	30	23.6%
Antimicrobial agents	13	10.2%
Mineral and ion-releasing agents	15	11.8%
Peptides and proteins	10	7.9%
β-Tricalcium phosphate	9	7.1%
Organomineral and natural-derived sources	6	4.7%
Functional coatings and carbon nanostructures	7	5.5%
Other	2	1.6%
Total	127	100%

**Table 5:** Classification of the included studies according to their level of biological validation.

Level of Biological Validation	Type of Biological Testing	Models Used	Number of Studies
In vitro	Cytotoxicity, adhesion, proliferation, ALP activity, osteogenic differentiation	MG-63, MC3T3-E1, hMSC, SaOS-2	67
In vivo	Bone formation, micro-CT, histology	Rat, mouse, rabbit, sheep	22
No reported biological testing	–	–	17

## Data Availability

The data supporting the findings of this study are derived from published literature. No new datasets were generated or analyzed in this study.

## Supplementary Materials

The following supporting information can be downloaded at: https://www.mdpi.com/article/10.3390/polym18030397/s1, Table S1. PRISMA 2020 checklist [141]; Table S2. Detailed search strategies used for literature retrieval in the Scopus, Web of Science.
