# Supplementary material for: Bioactive Polymer Composites for 3D-Printed Bone Implants: A Systematic Review
Source: Polymers (Basel). 2026 Feb 3;18(3):397. doi: 10.3390/polym18030397 (PMC12899301; doi:10.3390/polym18030397)
Supplement: Supplementary file 1 [file polymers-18-00397-s001.zip › Table S2. Detailed search strategies used for literature retrieval in the Scopus, Web of Science.pdf]

Table S2. Detailed search strategies used for literature retrieval in the Scopus, Web of Science

Database	Search date	Search fields	Complete search string	Limits applied
Scopus	15 September 2025	TITLE-ABS-KEY	`TITLE-ABS-KEY ( ("3D printing" OR "additive manufacturing") AND (bioactive OR osteoinductive OR antimicrobial) AND (bone OR "bone tissue engineering" OR "bone regeneration") AND (polymer OR composite OR "composite material" OR filament OR filaments) )`	Publication period: January 2016 – September 2025; Document type: Article; Language: English; Open access
Web of Science Core Collection	15 September 2025	Topic (TS)	`TS=( ("3D printing" OR "additive manufacturing") AND (bioactive OR osteoinductive OR antimicrobial) AND (bone OR "bone tissue engineering" OR "bone regeneration") AND (polymer OR composite OR "composite material" OR filament OR filaments) )`	Publication period: January 2016 – September 2025; Document type: Article; Language: English; Open access
